# Longitudinal study of MRI and functional outcome measures in facioscapulohumeral muscular dystrophy

**DOI:** 10.1186/s12891-021-04134-7

**Published:** 2021-03-10

**Authors:** Leo H. Wang, Dennis W. W. Shaw, Anna Faino, Christopher B. Budech, Leann M. Lewis, Jeffrey Statland, Katy Eichinger, Stephen J. Tapscott, Rabi N. Tawil, Seth D. Friedman

**Affiliations:** 1 Department of Neurology, University of Washington, Seattle, Washington USA; 2 Department of Radiology, University of Washington, Seattle, Washington USA; 3 Children’s Core for Biomedical Statistics, Seattle Children’s Research Institute, Seattle, Washington USA; 4 Department of Neurology, University of Rochester, Rochester, New York USA; 5 Department of Neurology, Kansas University Medical Center, Fairway, KS USA; 6 Human Biology Division, Fred Hutchinson Research Center, Seattle, Washington USA

**Keywords:** MRI, All neuromuscular disease, Muscle disease, Facioscapulohumeral muscular dystrophy (FSHD), Outcome measures

## Abstract

**Background:**

Facioscapulohumeral muscular dystrophy (FSHD) is a patchy and slowly progressive disease of skeletal muscle. For MRI to be a useful biomarker in an FSHD clinical trial, it should reliably detect changes over relatively short time-intervals (~ 1 year). We hypothesized that fatty change over the study course would be most likely in muscles already demonstrating disease progression, and that the degree of MRI burden would be correlated with function.

**Methods:**

We studied 36 patients with FSHD and lower-extremity weakness at baseline. Thirty-two patients returned in our 12-month longitudinal observational study. We analyzed DIXON MRI images of 16 lower-extremity muscles in each patient and compared them to quantitative strength measurement and ambulatory functional outcome measures.

**Results:**

There was a small shift to higher fat fractions in the summed muscle data for each patient, however individual muscles demonstrated much larger magnitudes of change. The greatest increase in fat fraction was observed in muscles having an intermediate fat replacement at baseline, with minimally (baseline fat fraction < 0.10) or severely (> 0.70) affected muscles less likely to progress. Functional outcome measures did not demonstrate marked change over the interval; however, overall MRI disease burden was correlated with functional outcome measures. Direct comparison of the tibialis anterior (TA) fat fraction and quantitative strength measurement showed a sigmoidal relationship, with steepest drop being when the muscle gets more than ~ 20% fatty replaced.

**Conclusions:**

Assessing MRI changes in 16 lower-extremity muscles across 1 year demonstrated that those muscles having an intermediate baseline fat fraction were more likely to progress. Ambulatory functional outcome measures are generally related to overall muscle MRI burden but remain unchanged in the short term. Quantitative strength measurement of the TA showed a steep loss of strength when more fatty infiltration is present suggesting that MRI may be preferable for following incremental change or modulation with drug therapy.

**Supplementary Information:**

The online version contains supplementary material available at 10.1186/s12891-021-04134-7.

## Background

Facioscapulohumeral muscular dystrophy (FSHD), the third most common muscular dystrophy, is heterogeneous in its presentation and progression of muscle weakness. The disease manifestation is variable but mostly slow in progression necessitating the identification and validation of more sensitive measures that can inform clinical trial efficacy in a time efficient manner.

Several studies have demonstrated that magnetic resonance imaging (MRI) measures can effectively follow FSHD disease progression over relatively short time scale. The primary finding is an approximately 2–5% fatty increase per year if a muscle progresses [1–4], with the suggestion that muscles showing moderate fatty infiltration and short tau inversion recovery (STIR) elevation at baseline are more likely to have a greater increase in fat fraction [4–7].

Studies to date have not demonstrated concordant MRI changes and decline in functional measures over 1 year [1, 2], in part because changes are small and can be highly variable. Loss of strength in single muscles were noted [1, 2] though uncorrelated to the magnitude of fatty progression. With many tasks, such as walking, involving many different muscle groups, there may be additional benefit in functionally summating total fat burden or determining a composite measure for evaluating change.

As part of our ongoing work studying muscle MRI and strength/functional measures longitudinally, we hypothesized that individual muscle MRI fat fraction would demonstrate change over the one-year interval, while the overall burden that is most related to functional outcome measure will not change over the same time period. We predict that the amount of fat fraction is associated with quantitative strength of the muscle. These data add to the growing collection of evidence that MRI measures of fatty replacement can observe progression in FSHD over the time-scales desired for most therapeutic trials, with functional outcome and strength measures adding critical context that may be further refined to better map onto the MRI results.

## Methods

We performed a prospective, longitudinal, observational study of FSHD patients through the Seattle Paul D. Wellstone Muscular Dystrophy Cooperative Research Center at the University of Rochester (UR) and University of Washington (UW) from 2015 to 2017.

### Participants

A total of 36 patients were recruited (18 at UW and 18 at UR) through clinic visits and the UR National Registry for Myotonic Dystrophy and FSHD. All patients had genetically confirmed FSHD1 and/or 2. Thirty-six patients at baseline and 32 patients at follow-up were examined using functional outcome measures and MRI of the lower extremities.

### Standard protocol approvals, registrations, and patient consents

The study was approved by the Human Subjects Committee at each institution, with written informed consent obtained for all participants.

### Clinical assessments

Clinical assessments included Clinical Severity Score (CSS, a validated, 10-grade overall clinical severity scale; 0 = unaffected, 10 = wheelchair dependent), and quantitative strength measurement (in Newton-force) of the tibialis anterior (TA) using a hand-held dynamometer [8, 9]. Two established leg functional outcomes to assess lower extremity function were performed: 6-min walk test (6MWT) and go-30-ft (go 30′). 6MWT is a test of functional exercise capacity that measures the distance an individual can walk on a level surface in 6 min. Go 30′ measures the time an individual can traverse 30 ft as quickly as possible.

### Muscle magnetic resonance imaging

All MRI examinations were performed on 3T Siemens PRISMA scanners running software E11C. Sequences were acquired using flexible array coils (to acquire both thigh and calf in the bilateral legs) with the following parameters: 3-plane localizers; T1 (TE = 8.9 ms, TR = 510 ms, 320 × 224, 5 mm thick, 40 slices); STIR (same resolution, TE = 38 ms, TR = 4790 ms, TI = 220 ms, flip 150°); a three-echo fat-saturated T2 series (TE = 13.2, 26.4, 39.6 ms, TR = 1450 ms, 5 mm thick, 40 slices, matrix = 320 × 320); and a multi-echo DIXON sequence (TE = 1.3, 2.48, 3.73, 4.98, 7.38, 9.84 ms, TR = 11.3 ms, 5 mm thick). Representative T1 and STIR images and examples changing over time are presented in Supplemental Figure 1.

### Muscle MRI quantitative analysis

STIR intensity were rated qualitatively (by DWS and SDF) on a four-point scale: 0, normal appearance; 1, very mild diffuse increase/may be artifact, close to zero; 2, mild diffuse increase; 3, moderate areas of increased signal intensity; 4, severe involvement of entire muscle as per our previous work [10]. STIR positivity appears to denote active disease state as assessed by DUX4-targeted expression in STIR positive muscle in FSHD patients [10].

Multi-level DIXON analysis: Dixon data was imported into ITK-snap (Version 3.6.0) for individual muscle labelling (sartorius, vastus lateralis, semimembranosus, tibialis anterior, tibialis posterior, soleus, medial gastrocnemius, and lateral gastrocnemius) on the raw DIXON opposing phase image, owing to the ease of conspicuity of muscle borders. These regions were chosen for parallel work (MRI-biopsy association [10, 11]) and to survey a “control” region that is rarely affected (tibialis posterior). A continuous series of axial images of approximately 100 mm in the center of the calf and proximal thigh was selected for segmentation, involving tracing every third slice and interpolating, then manual cleaning up of the result. Conservative muscle borders were drawn to avoid sub-cutaneous fat on the outer edge and shared muscle pixel boundaries and connective tissue between muscle bodies. A first pass of verification checked the anatomical extent of each mask by a secondary rater. A similar process was performed blindly for follow-up data. A final check-step involved a single rater identifying the matched series of slices between baseline and follow-up data for statistical comparison. This yielded an approximately 80 mm cranial-caudal segment of calf and thigh. The fat fraction per muscle was the average of the multiple slices sampled. Automated calculation of fat fraction was then performed in MATLAB (Mathworks 2018a, Natick MA) with ITK-masks integrated with water- and fat-derived maps.

### Muscle fat fraction burden index

The proximal anterior (PA) compartment was represented by the sartorius and vastus lateralis; proximal posterior (PP) by the semimembranosus; distal anterior (DA) by the tibialis anterior (TA); and distal posterior (DP) by the tibialis posterior (TP), soleus, medial gastrocnemius (MG), and lateral gastrocnemius (LG). The Muscle Fat Fraction Burden Index was calculated as a sum of the four compartments—16 leg muscles (8 in each leg).

### Statistical analyses

Descriptive summaries, scatterplots and histograms were used to examine muscle fat fractions and functional outcome measures at baseline and follow up. In addition, changes in muscle fat fraction and changes in functional outcome measures between baseline and follow up were summarized both individually in scatterplots and boxplots, and together in correlation plots. Fat fractions at baseline, follow up and the change between baseline and follow up were summarized using median with interquartile range (if data were non-parametric), mean with standard deviation, and also by 0.10 fat fraction bins (fat fraction deciles). A coefficient of variation was calculated for the fat fraction change in individual muscles. Assuming progression of disease results in positive changes only in fat fraction, we estimated the error in the DIXON quantitative measurement based on the range of negative fat fractions, and defined a meaningful change as at or above this ± error threshold.

In order to assess whether or not the fat fraction scores significantly changed over time, a linear mixed effect model (LMEM) was used to regress the Muscle Fat Fraction Burden Index, along with the four leg compartments separately, on visit (baseline or follow up), where visit was the independent variable in the model. In order to assess whether or not there was an association between functional outcome measures and fat fraction scores, 6MWT distance and go 30′ time were regressed on the following independent variables using LMEMs: visit, fat fraction, and the interaction between visit and fat fraction. If the interaction between visit and fat fraction was not significant, it was removed from the model. For all LMEMs, the correlation between multiple observations within participant was accounted for by specifying a random intercept per participant in the LMEM. In addition, potential study site differences (at UW or UR) were accounted for by specifying a random intercept per study site in all LMEMs.

For LMEMs with 6MWT and Go 30′ time as the outcome of interest, we were interested in comparing results from three models: (a) modeling each of the eight muscles as independent variables separately in one single model, (b) modeling the four compartments as independent variables separately in one single model, and (c) modeling the Muscle Fat Fraction Burden Index as one single independent variable. Model goodness-of-fit was compared between models (b) and (c) using Akaike Information Criterion (AIC), where a lower AIC indicated better model fit.

For quantitative strength analyses, we examined the relationship between TA fat fraction and strength using a scatterplot with locally estimated scatterplot smoothing (LOESS). Other comparisons, such as whether STIR positive status at baseline related to the degree of fat fraction at baseline or the degree of fat fraction change from baseline to follow up, were summarized using descriptive statistics and plots. A two-sided test *p*-value less than 0.05 was considered statistically significant, and all analyses were completed in R version 3.5.1.

## Results

### Study cohort

Thirty-six patients were recruited, of which 34% were female, with age range of 20–75 years (median 57 years) and Clinical Severity Score range of 0–9 (median 5.5). The patient demographics, genotype, disease severity, and lower extremity functional outcome measures are presented in Supplemental Table 1. All patients were confirmed FSHD1 except two patients with FSHD2 and another patient with mosaic FSHD1. All except one returned 12 months (average 378 days) later.

### Quantitative fat fraction at baseline and 12-month follow-up

**Fig. 1:**
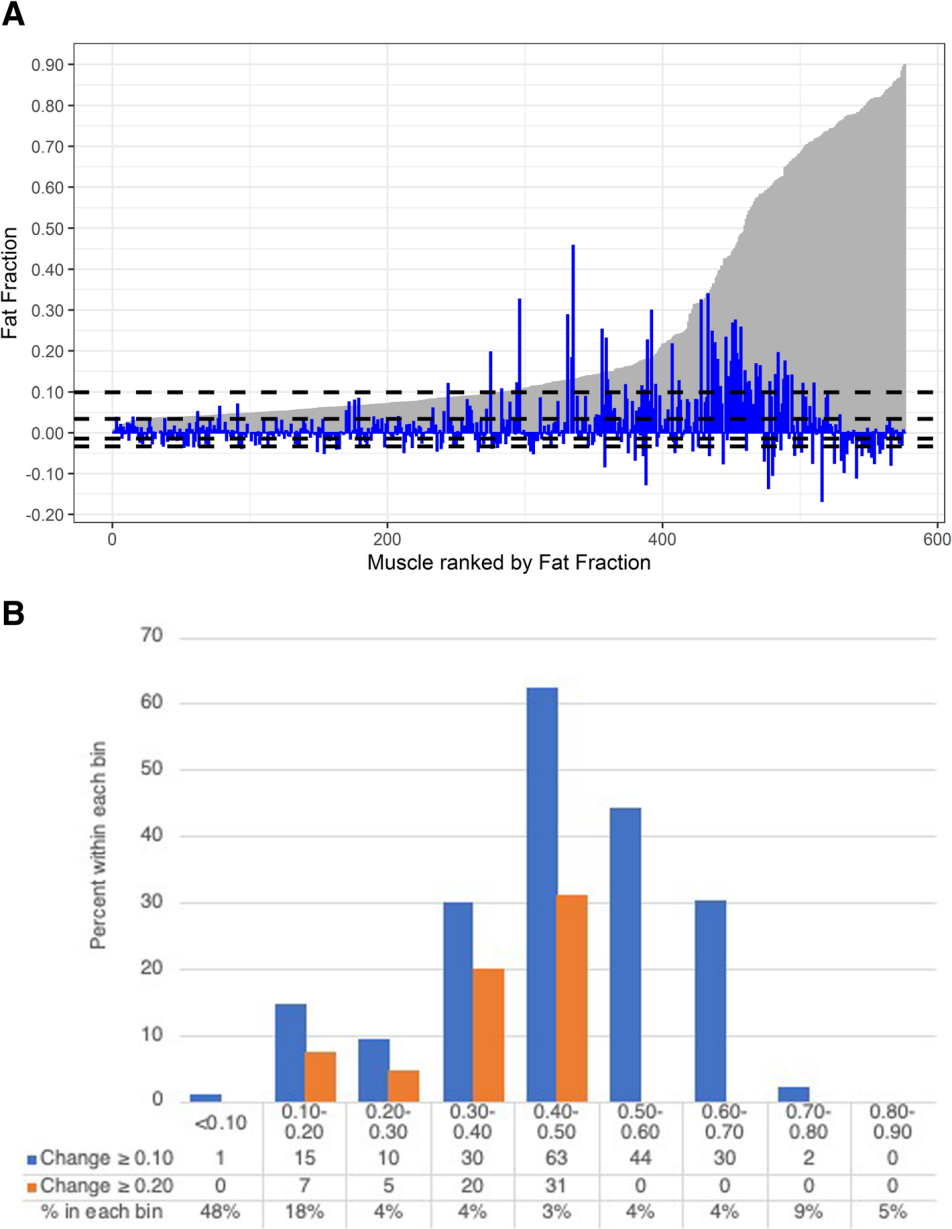
**a** Barplot of baseline muscle fat fractions (in gray) with overlay of change in fat fractions (in blue). Horizontal dotted lines at % change 10th, 25th, 75th and 90th percentiles (values of − 0.03, − 0.01, 0.03 and 0.10). 50th and 75th percentiles for baseline fat fraction were 0.10 and 0.34, respectively. **b** Percentage of muscles within each 10% bin at baseline that have an increase of greater than 0.1 or 0.2 at follow-up

Multi-level DIXON quantitative measurements were performed on eight muscles over multiple sample slices in each leg, in 36 patients at baseline and 32 patients at follow-up. As a population of individual muscles, a small change in fatty infiltration was observed over 1 year; the median [IQR] change in fat fraction was 0.01 [− 0.01, 0.03] (mean ± SD: 0.02 ± 0.07). Given a coefficient of variance of 3.5 for change in fat fraction over 1 year (with a signal-to-noise ratio of 1:3.5). Of all 576 muscles assessed at baseline by DIXON quantitative measurement, half of the muscles had fat fraction less than 0.10 (Fig. 1a). The semimembranosus and medial gastrocnemius were the most affected with greater fat infiltration at baseline (Table 1), with median [IQR] fat fractions of 0.33 [0.06–0.74] and 0.21 [0.07–0.70], respectively.

**Table 1:** The median [IQR] fat fraction at baseline and follow-up in each muscle

	Baseline	Follow-up	Difference
Sartorius	0.14 [0.09,0.23]	0.18 [0.10,0.26]	0.02 [0,0.5]
Vastus lateralis	0.10 [0.07,0.19]	0.11 [0.07,0.29]	0.02 [0,0.05]
Semimembranosus	0.33 [0.06,0.74]	0.65 [0.11,0.76]	0.01 [0,0.07]
Tibialis anterior	0.09 [0.05,0.56]	0.10 [0.04,0.64]	0 [− 0.03,0.02]
Tibialis posterior	0.06 [0.04,0.09]	0.06 [0.04,0.10]	0 [− 0.02,0]
Soleus	0.10 [0.06,0.24]	0.11 [0.07,0.40]	0 [− 0.02,0.03]
MG	0.21 [0.07,0.70]	0.24 [0.08,0.76]	0 [0,0.05]
LG	0.09 [0.05,0.19]	0.09 [0.05,0.31]	0 [−0.03,0.04]

**Fig. 2:**
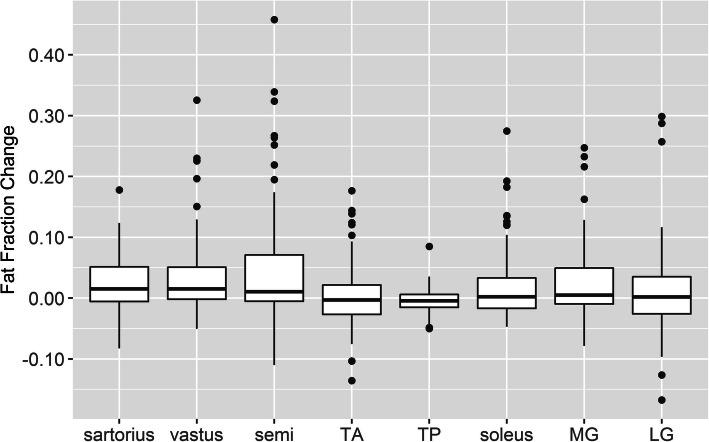
Boxplot of Change in muscle fat fraction

In specific muscles, the sartorius, vastus lateralis, and semimembranosus had a median increase of 0.01–0.02 (1–2%) in fat fraction after 12 months (Table 1 and Fig. 2). The semimembranosus demonstrated the most change: 20% (14/68) of muscles increased by 0.10 in fat fraction and 12% (8/68) increased by 0.20. Among all muscles examined, the greatest single-most fat fraction change was seen in the right semimembranosus of patient 32–009, increasing from 0.13 to 0.59 (increase of 0.46); in contrast, the contralateral muscle of this individual increased 0.66 to 0.80 (increase of 0.14). Across all patients, minimal changes (median change centered around 0 over 12 months) were found in the tibialis anterior, tibialis posterior, soleus, medial and lateral gastrocnemius (Table 1 and Fig. 2).

As exploration, we divided muscles by their baseline fat fraction into 0.10 fat fraction bins, and if we utilize our definition of minimal significant change as an increase of ≥0.10, the highest percentage of changes were seen in muscles with 0.40–0.50 baseline fat fraction—where 63% of muscles increased by ≥0.10; no muscles changed ≥0.10 once the baseline muscle fat fraction was more than 0.80 (Fig. 1b). Higher magnitude changes peaked at 0.40–0.50, with no muscles with baseline fat fraction above 0.50 increasing ≥0.20.

### Assessment of change in muscle fat fraction burden index

We calculated a Muscle Fat Fraction Burden Index summating the fat fraction of the 16 muscles we measured. We found a significant change in the Muscle Fat Fraction Burden Index longitudinally (model estimate: 0.35, 95% CI [0.15, 0.54], Table 2). Over 12 months, the median [IQR] change in the Muscle Fat Fraction Burden Index (of the 32 patients with follow-up MRI scans) was 0.24 [0.07, 0.63] (mean ± SD: 0.34 ± 0.55). When examining change within each muscle compartment, we found significant longitudinal changes in the DP, PA and PP leg compartments, and no significant change longitudinally for the DA leg compartment.

**Table 2:** LMEM results, change in muscle fat fraction scores from baseline to follow-up. Separate models run for each leg compartment fat fraction score, where beta coefficient corresponds to time from baseline to follow-up

Muscle fat fraction score	Beta [95% CI]	***P***-value	Reference:	
			Median [IQR] Score at baseline	IQR change in Score from baseline to follow-up
Muscle Fat Fraction Burden Index	0.35 [0.15, 0.54]	<.001	2.90 [1.54, 5.79]	[−0.12, 0.62]
DA leg compartment score	0.00 [−0.03, 0.04]	0.78	0.16 [0.09, 1.14]	[−0.06, 0.02]
DP leg compartment score	0.12 [0.01, 0.22]	0.026	1.00 [0.48, 2.74]	[−0.15, 0.20]
PA leg compartment score	0.12 [0.06, 0.18]	<.001	0.55 [0.34, 0.92]	[−0.03, 0.18]
PP leg compartment score	0.11 [0.04, 0.18]	0.001	0.76 [0.14, 1.45]	[−0.03, 0.10]

### STIR intensity correlates with fat fraction increase over 12 months

**Fig. 3:**
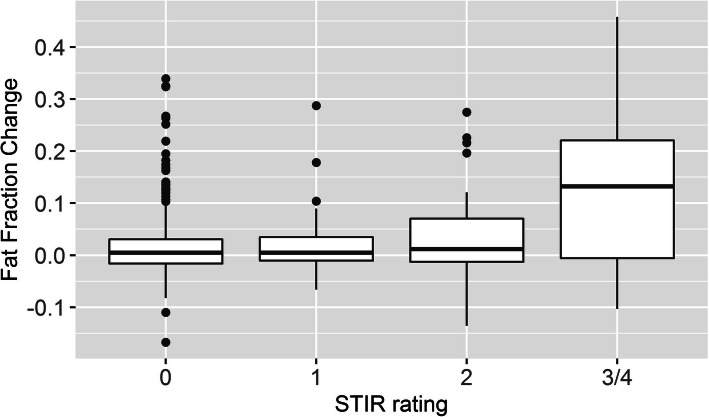
Boxplot of change in fat fraction by STIR rating

We performed a secondary analysis to determine whether STIR positivity portended changes in fat fraction on MRI. All 512 muscles with fat fraction data at baseline and 12-month follow-up were graded by STIR hyperintensity at baseline. The gastrocnemii followed by the vastus lateralis and tibialis anterior, equally, had the most STIR positive muscles (see Supplemental Table 2). Most of the muscles (78%) were STIR negative covering a wide range of T1 fat fraction at baseline—median [IQR] 0.10 [0.06, 0.54] (supplemental Figure 2). Muscles with STIR 1 and 2 hyperintensity levels had similar median baseline fat fraction (0.09–0.10, Table 3, Fig. 3 and supplemental Figure 2). In contrast, higher STIR hyperintensity (3 and 4) was associated with higher baseline T1 fat fraction (median [IQR]: 0.17 [0.09, 0.45]).

**Table 3:** Median fat fraction at baseline and change after 12 months based on STIR rating

STIR	Median [IQR] fat fraction at baseline	Median [IQR] fat fraction change	Number
0	0.10 [0.06,0.54]	0.005 [−0.02,0.03]	399
1	0.09 [0.05,0.17]	0.005 [−0.01,0.03]	54
2	0.10 [0.05,0.31]	0.012 [−0.01,0.07]	37
3/4	0.17 [0.09,0.45]	0.12 [0,0.21]	23

More importantly, higher STIR intensity was associated with higher increase in fat fraction over 12 months. While the median change over 12 months at STIR = 0 or 1 was 0.005, the change was more than an order of magnitude higher for STIR = 3 or 4 at 0.12 (see Table 3 and Fig. 3); however, the change spanned a large distribution with IQR of STIR = 3 or 4 between 0 and 0.21.

### Association of quantitative fat fraction with functional outcome measures

**Fig. 4:**
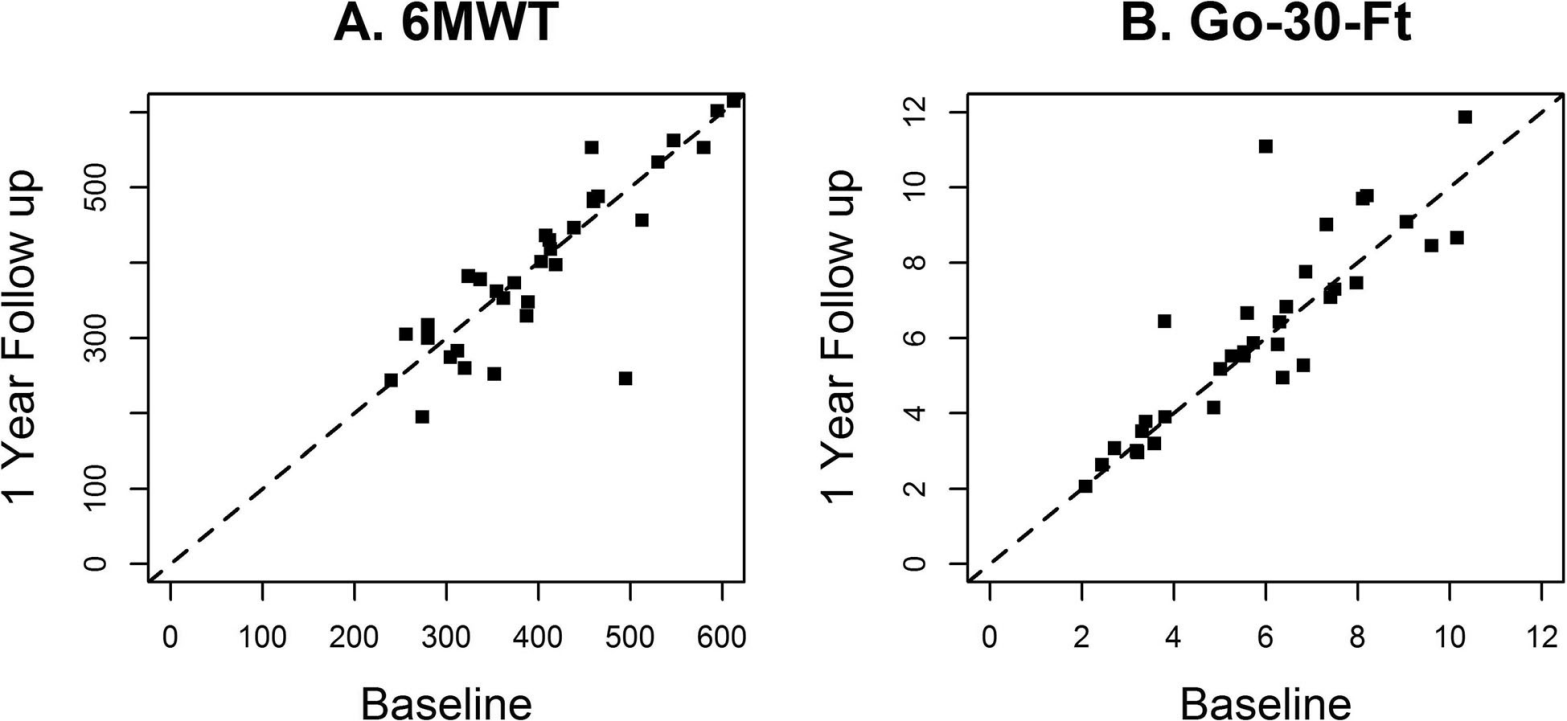
Ambulatory functional outcome measures over 12-month period. Comparing baseline (x-axis) and 1-year follow-up (y-axis) of 6MWT (m) and go-30-ft (seconds)

The median 6MWT at baseline was 403 m, with interquartile range [IQR] spanning 322 to 463 m. Over 12 months, there was no appreciable change in the 6MWT: median [IQR] increase was 4 [− 30, + 21] meters; the distribution suggested variable changes that crossed zero. Go 30′ at baseline showed a median [IQR] of 6.0 [3.8, 7.5] seconds; at follow-up, the change was minimal with a median [IQR] change of + 0.1 [− 0.3, + 0.4] seconds. Figures 4a and b compare 6MWT and Go 30′ at baseline and follow-up.

Increases in total Muscle Fat Fraction Burden Index, as well as a measure we derived of the distal posterior (DP) leg compartment score (medial gastrocnemius, lateral gastrocnemius and soleus), were associated with a decrease in 6MWT and an increase in Go 30′ (Tables 4 and 5). Changes in individual muscle fat fractions were not significantly associated with 6MWT or Go 30′ (Supplemental Table 3). While AIC improved when regressing 6MWT on the four individual leg compartments versus the Muscle Fat Fraction Burden index, the opposite was true for Go 30′.

**Table 4:** LMEM results, 6MWT and Go-30-Feet regressed on visit and Muscle Fat Fraction Burden Index. Fat burden index coefficient corresponds to an IQR change increase

Variable	6MWT (AIC = 710.54)		Go-30-Feet (AIC = 253.60)	
	Beta [95% CI]	***P***-value	Beta [95% CI]	***P***-value
Intercept	495.5 [448.1, 543.7]	<.001	4.32 [3.18, 5.51]	<.001
Follow-up vs baseline	−7.66 [−32.36, 17.35]	0.54	0.16 [−0.34, 0.65]	0.51
Muscle Fat Fraction Burden Index	−18.63 [−26.20, −11.04]	<.001	0.37 [0.18, 0.55]	<.001

**Table 5:** LMEM results, 6MWT and Go-30-Feet regressed on visit and leg compartment scores. Subsection score coefficients correspond to IQR change increases

Variable	6MWT (AIC = 698.91)		Go-30-Feet (AIC = 265.26)	
	Beta [95% CI]	***P***-value	Beta [95% CI]	***P***-value
Intercept	495.7 [445.1, 546.2]	<.001	4.47 [3.20, 5.75]	<.001
Follow-up vs baseline	−8.6 [−34.1, 16.6]	0.51	0.22 [−0.30, 0.72]	0.39
DA leg compartment score	2.06 [−5.15, 9.30]	0.59	−0.03 [− 0.21, 0.15]	0.76
DP leg compartment score	−19.46 [−30.94, −7.97]	0.001	0.39 [0.10, 0.69]	0.009
PA leg compartment score	1.44 [−11.91, 14.76]	0.84	−0.03 [− 0.35, 0.31]	0.88
PP leg compartment score	−3.29 [−9.78, 3.26]	0.34	0.02 [−0.13, 0.18]	0.80

### TA fat fraction correlated with quantitative strength measurements

**Fig. 5:**
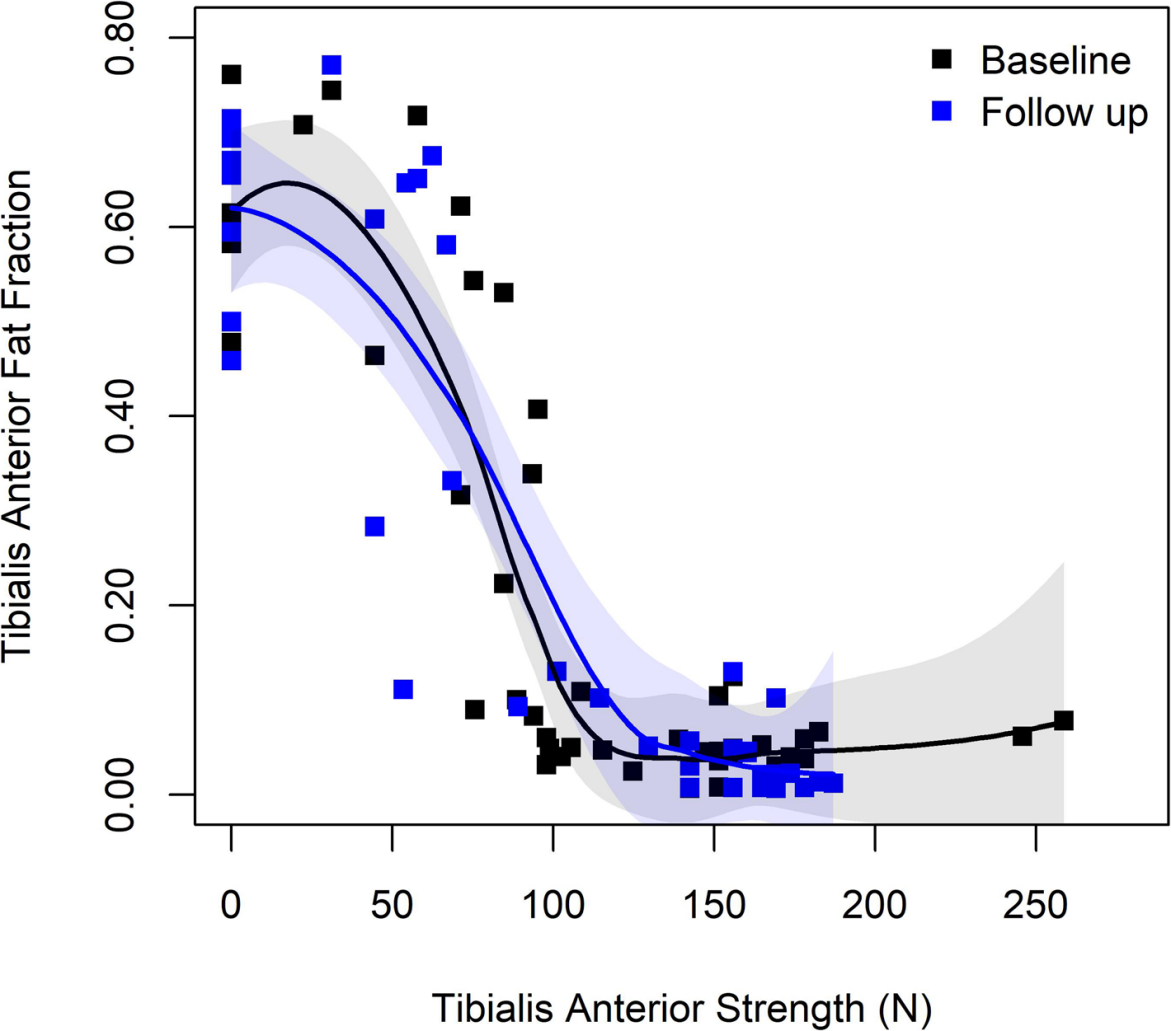
Scatterplot of fat fraction versus tibialis anterior strength at both time points. LOESS curves with 95% confidence intervals plotted separately for baseline and follow-up time points

Compared to outcome measures consisting of complex motions, foot dorsiflexion strength measured by quantitative handheld dynamometer maps directly onto the function of the TA. Foot dorsiflexion strength was measured for 47 legs (24 patients) at baseline and 39 legs (21 patients) at follow-up. There was a sigmoidal relationship between the amount of fatty infiltrate and the strength of the TA at baseline. This sigmoidal relationship remained at follow-up, without sufficient numbers of patients exhibiting TA change to demonstrate association. The two curves showed a floor and ceiling effect—most of the muscles with fat fraction below 0.10 associated with greater than 90 Newtons of strength while fat fraction above 0.40 associated with less than 67 Newtons of strength (Fig. 5).

## Discussion

In this paper, we present the longitudinal follow-up to our original cohort of 36 patients who had lower-extremity MRI, muscle biopsies, and functional outcome measures at baseline [10]. Our goal was to add to the collective literature evaluating FSHD muscle evolution over 12 months and examine how MRI changes best map onto changes in behavioral performance. Findings demonstrate while the overall T1 fat burden is associated with function, sub-groupings of muscles better explained task variance for specialized ambulation (as an example). While more work is needed to determine if other muscles have a similar sigmoidal relationship between fat fraction and strength as the TA, fatty progression within individual muscles is perhaps the best biomarker to try and halt or slow with therapeutic intervention.

### T1 fat fraction at baseline and 12-month follow-up

Muscle structural integrity was assessed on T1 imaging and quantitated with multi-slice DIXON fat quantification. We found that slightly less than half of the muscles examined at baseline were less than 0.10 fatty infiltrated similar to the findings of Mul et al. [12]; and approximately less than a quarter of the muscles examined were more than 0.50 fatty infiltrated. We also found that the hamstring (semimembranosus) was the most fatty-infiltrated and the medial gastrocnemius to be the second most fatty infiltrated, similar to a large set of prior reports [7, 12–14]. Our finding that muscles with a baseline fat fraction less than 0.10 or greater than 0.80 are less likely to progress significantly in fat fraction over a 12-month interval is similar to findings in Andersen et al. and Dahlqvist et al. [1, 5]. In our cohort, the highest percentage of measurable change peaked in those muscles having intermediate fat fraction of ~ 0.40–0.50 at baseline; with large changes in fat fraction (≥ 0.20) only seen in less affected muscles. This has been described in other muscular dystrophies such as Duchenne muscular dystrophy [15, 16].

### Correlation of STIR hyperintensity with T1 fat fraction increase

There was variable STIR hyperintensity amongst those muscles that transformed and those that remained stable. Similar to the results reported in Dahlqvist et al. [5], we also saw some larger magnitude of fatty progression in those STIR muscles that were rated as more severe in brightness and extent. However, progression of fatty infiltration was also measured in muscles without visible STIR hyperintensity. Understanding the nature of what disease processes cause STIR hyperintensity, and how these changes fit into the overall decline seen in FSHD muscles, remains fodder for future work.

### MRI changes and association with functional outcome measures

Leg fat fraction was associated with decreased lower-extremity functional outcome. These results align with prior reports of Leung et al. [13] which found that hamstring mean fatty infiltrative score by semi-quantitative visual analysis correlated with the 10-m walk test and Mul et al. [12] which found a correlation of mean fat fraction of total leg musculature with 6MWT. Similar results have been shown in Duchenne muscular dystrophy with single voxel 1H MR spectroscopy quantification of intramuscular fat fraction in the vastus lateralis correlated with the 6MWT [17, 18].

In an exploratory context, creating fat fraction indexes by functional compartments were more closely associated with complex motor performance like walking than simple summation. At face-value this makes intuitive sense since 100% fatty progression in a medial gastrocnemius will have significantly less impact on walking that having a fully progressed hamstring. While various tasks may require different groupings, and there are many other features that could be integrated into a model (e.g., asymmetry, retained muscle volume, fraction of STIR-hyperintense volume over retained muscle volume, or fat/muscle fraction, as well as other modulators, such as how upper body posture affects walking), this initial data provide a conceptual starting point to think about how to best map MRI metrics from multiple muscles onto complex task performance.

### Association between TA fat fraction and quantitative strength measurement

The “goal-state,” where near-perfect overlap is found between task performance and the muscle subserving that function, can be seen in our exploratory data between TA fat fraction and quantitative strength. Muscle strength has been found to be correlated with muscle fat fraction area in knee flexors and extensors [5, 7]. Other attempts have been in semi-quantitative fashion: Leung et al. [13] found that quantitative muscle strength measurements of shoulder abduction, arm flexion, hip flexion, and knee extension/flexion correlated with a semi-quantitative visual scale of T1 fat infiltration. Olsen et al. [14] and Kan et al. [19] in smaller cohorts (*N* = 18 and 8 respectively) found that categorical Medical Research Council strength score inversely correlated with muscle fat fraction.

In our data, the association between foot dorsiflexion strength and TA fat fraction was sigmoidal, with relative preservation of strength with fat fraction levels < 0.10, and steep fall off thereafter. Overall, our findings suggest that muscles with fat fraction > 0.20 have significant weakness. If other muscles follow this specific pattern, it would mean that a good goal for a clinical treatment trial would be prevention of progression above this threshold. Future work will be helpful to define the relationship between fat fraction and strength of other muscles to confirm the generalizability of our TA findings.

### Limitations and further thoughts

Finally, several points warrant mention: Though we evaluated a larger region of interest than some studies [12], we did not scan muscles from their full proximal to distal extent. This may have underestimated our assessment of fat fraction change, since some investigations have observed a proximal to distal gradient in fat infiltration, though other studies have found relatively uniform change over a 14-month period across the individual muscles despite the baseline greater fat infiltration distally [5, 7]. Further, while we verified the extent of the slabs between the baseline- and follow-up exams, and used conservative regions-of-interest toward measuring unambiguous fat/muscle fractions, the presence of negative fat fraction changes in some muscles, similar to other studies [1, 5], suggests that acquisition and analytic improvements remain to made.

One option for improved and unambiguous muscle/fat fraction measurement could be 1H spectroscopy [18], though the trade-off is acquisition time and spatial resolution. A further limitation of MRS is the challenge of discriminating similar water components (normal muscle, STIR+). While some hybrid (imaging+MRS) approach could certainly be used, given some biopsy samples from normal T1/STIR appearing tissue can show histologic abnormalities [10] or be energetically compromised [19], there remains more work to be done to determine if other biomarkers might provide additional explanatory variance (ultra-short TE-imaging [20] might provide a way to assess MRI signal changes indicative of other pathological features, such as fibrosis).

## Conclusions

This study demonstrates the sensitivity of leg fat-fraction as a biomarker of FSHD progression over a 1-year interval, adding to the accumulated data from other studies. While summation of fat fraction in the lower extremity demonstrates change over a year, the small magnitude of change masks the progression that is observed by individual muscles. Consistent with the literature, individual muscles with a higher probability of progression often demonstrate an intermediate fat fraction and STIR-hyperintensity at baseline. Creating compartmental summaries of fat fractions may be the best approach to map data onto functional outcome measures, of critical importance to measure for change over long-term study intervals. What is clear from our specific TA results is that a muscle with intermediate fat fraction may already be functionally weak. This suggests that while you can measure small fractional changes in fat with MRI over time, that strength and functional outcome measures might lag behind and/or perhaps advance in a rapid fashion when some fractional threshold is crossed. If true, has implications for clinical trial design and measurement, with the goal of keeping muscles above specific thresholds being the treatment target.

## Supplementary Information

**Additional file 1: Figure S1.** Longitudinal T1 and STIR images are shown from two patients over the study interval. At top (patient 32-016), fatty replacement is seen within the medial gastrocnemius and soleus. Both muscles retain STIR+ signal in non-fatty replaced regions foreshadowing likely progression. At bottom (patient 32-006), a localized STIR bright region in the extensor digitorum is seen at baseline. This region is fatty replaced at the 1-year follow-up.

**Additional file 2: Figure S2.** Barplot of baseline muscle fat fractions (in gray) with overlay of change in fat fractions (in blue) by baseline STIR rating. Horizontal dotted lines at % change 10th, 25th, 75th and 90th percentiles (values of -0.03, -0.01, 0.03 and 0.10).

**Additional file 3: Figure S3.** ITK snap workflow that has loaded the raw in-phase and out-phase DIXON images and the derived fat and water maps. Muscles of interest were traced on a slice by slice basis to encompass a central matched slab across the muscle body. The interface allows rapid switching between raw data series to determine the boundaries of the muscles labeled. At top, right, and bottom right, overlays are shown, with derived 3D volumes at bottom left.

**Additional file 4: Table S1.** Patient demographics, genotype, and functional outcome measures. **Table S2.** STIR-positive muscles by baseline STIR hyperintensity levels. **Table S3.** LMEM results, 6MWT and Go-30-Feet regressed on visit and individual muscle fat fractions. Fat fraction coefficients correspond to IQR change increases.

## Data Availability

The datasets used and/or analysed during the current study are available from the corresponding author on reasonable request.

## Abbreviations

FSHD: Facioscapulohumeral muscular dystrophy
MRI: Magnetic resonance imaging
STIR: Short tau inversion recovery
IQR: Interquartile range
CSS: Clinical Severity Score
6MWT: 6-min walk test
Go 30′: Go-30-ft
TUG: Time-up-and-go
PA: Proximal anterior compartment
PP: Proximal posterior
DA: Distal anterior (DA)
DP: Distal posterior (DP)
TA: Tibialis anterior
TP: Tibialis posterior
MG: Medial gastrocnemius
LG: Lateral gastrocnemius
LMEM: Linear mixed effect model
AIC: Akaike Information Criterion (AIC)
LOESS: Locally estimated scatterplot smoothing (LOESS)
