# Supplementary figures and images for: Longitudinal study of MRI and functional outcome measures in facioscapulohumeral muscular dystrophy
Source: BMC Musculoskelet Disord. 2021 Mar 10;22:262. doi: 10.1186/s12891-021-04134-7 (PMC7948347; doi:10.1186/s12891-021-04134-7)

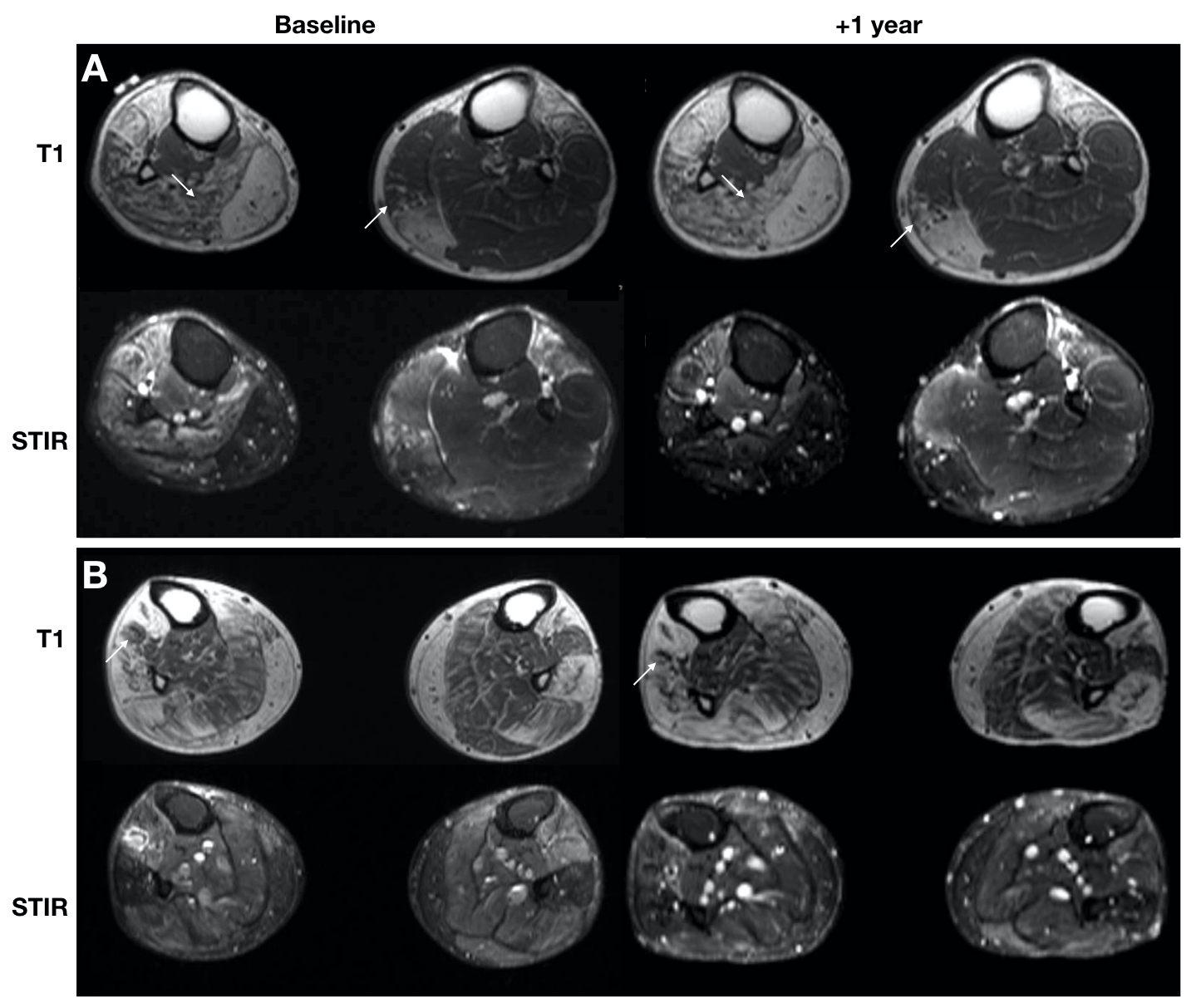

Supplement: Supplementary file 1 — Additional file 1: Figure S1. Longitudinal T1 and STIR images are shown from two patients over the study interval. At top (patient 32-016), fatty replacement is seen within the medial gastrocnemius and soleus. Both muscles retain STIR+ signal in non-fatty replaced regions foreshadowing likely progression. At bottom (patient 32-006), a localized STIR bright region in the extensor digitorum is seen at baseline. This region is fatty replaced at the 1-year follow-up. [file 12891_2021_4134_MOESM1_ESM.jpeg]

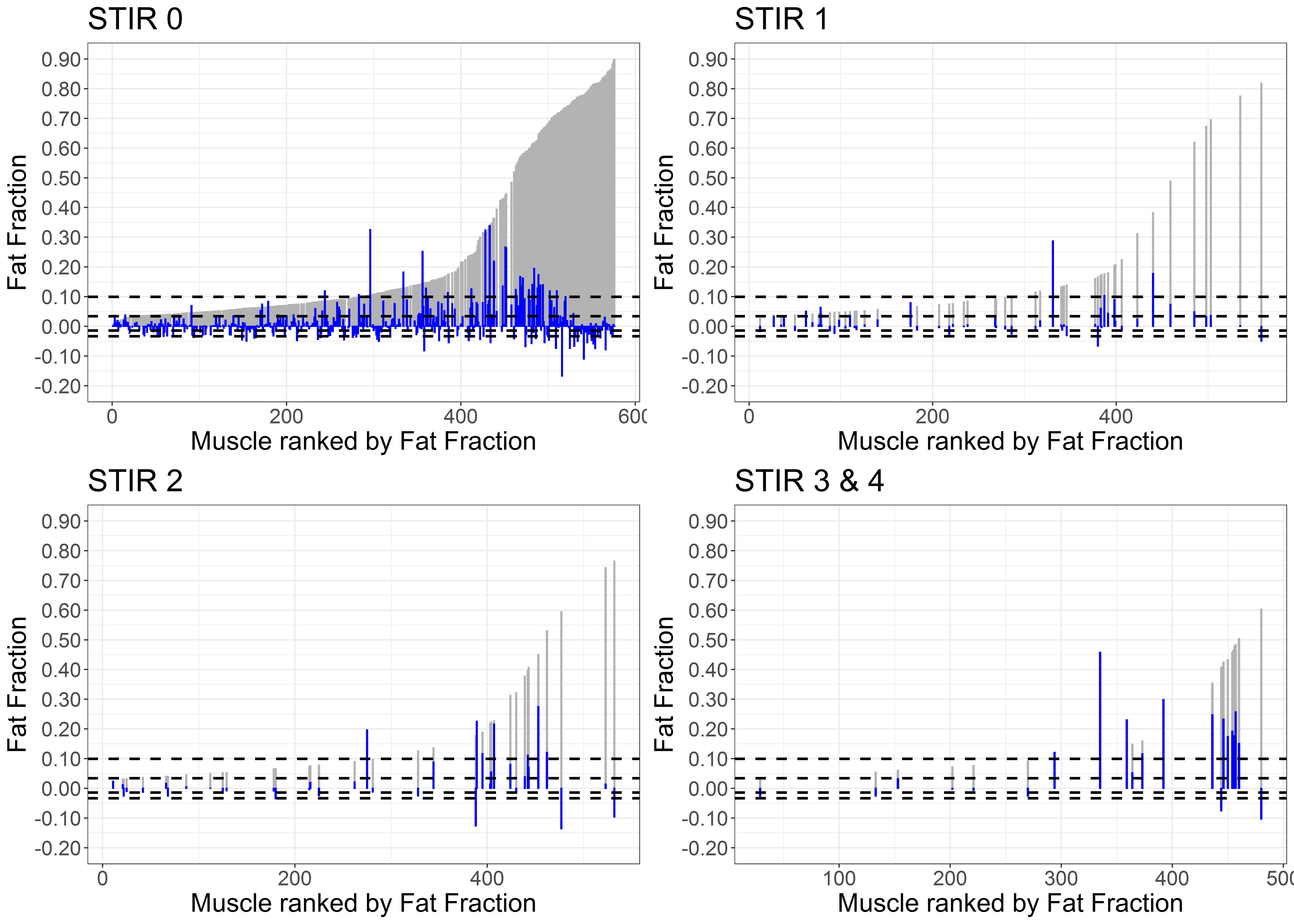

Supplement: Supplementary file 2 — Additional file 2: Figure S2. Barplot of baseline muscle fat fractions (in gray) with overlay of change in fat fractions (in blue) by baseline STIR rating. Horizontal dotted lines at % change 10th, 25th, 75th and 90th percentiles (values of -0.03, -0.01, 0.03 and 0.10). [file 12891_2021_4134_MOESM2_ESM.jpg]

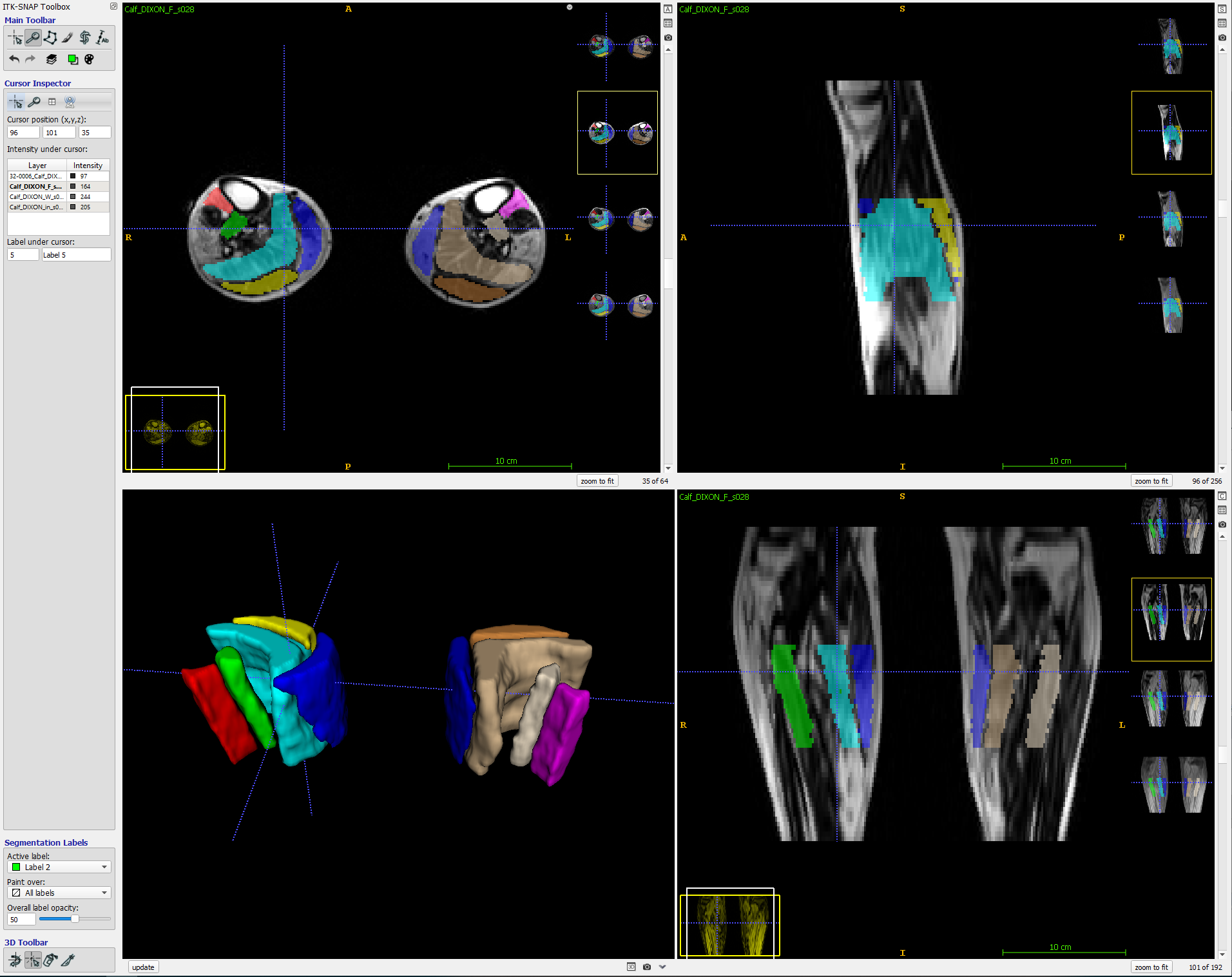

Supplement: Supplementary file 3 — Additional file 3: Figure S3. ITK snap workflow that has loaded the raw in-phase and out-phase DIXON images and the derived fat and water maps. Muscles of interest were traced on a slice by slice basis to encompass a central matched slab across the muscle body. The interface allows rapid switching between raw data series to determine the boundaries of the muscles labeled. At top, right, and bottom right, overlays are shown, with derived 3D volumes at bottom left. [file 12891_2021_4134_MOESM3_ESM.tiff]
