# Supplementary material for: Longitudinal study of MRI and functional outcome measures in facioscapulohumeral muscular dystrophy
Source: BMC Musculoskelet Disord. 2021 Mar 10;22:262. doi: 10.1186/s12891-021-04134-7 (PMC7948347; doi:10.1186/s12891-021-04134-7)
Supplement: Supplementary file 4 — Additional file 4: Table S1. Patient demographics, genotype, and functional outcome measures. Table S2. STIR-positive muscles by baseline STIR hyperintensity levels. Table S3. LMEM results, 6MWT and Go-30-Feet regressed on visit and individual muscle fat fractions. Fat fraction coefficients correspond to IQR change increases. [file 12891_2021_4134_MOESM4_ESM.docx]

**Supplemental Tables**

**Supplemental Table 1. Patient demographics, genotype, and functional outcome measures**

| **Subject #** | **Age** | **Age of onset** | **Sex** | **CSS** | **Short4q (kb)** | **6MWT** | | **Go-30-feet** | |
| --- | --- | --- | --- | --- | --- | --- | --- | --- | --- |
|  |  |  |  |  |  | **Year 1** | **Year 2** | **Year 1** | **Year 2** |
| 01-0022 | 56 | 21 | M | 5 | 18 | 240 | 244 | 8.0 | 7.5 |
| 01-0023 | 54 | 30 | F | 6 | 25 | 280 | 300 | 7.4 | 7.1 |
| 01-0024 | 49 | 10 | M | 4 | 26 | 595 | 602 | 3.2 | 3.0 |
| 01-0025 | 56 | 10 | M | 3 | 90* | 530 | 534 | 3.6 | 3.2 |
| 01-0026 | 60 | 35 | M | 7 | 30 | 304 | 275 | 8.2 | 9.8 |
| 01-0027 | 60 | 15 | M | 8 | 22 | 312 | 283 | 9.1 | 9.1 |
| 01-0028 | 57 | 33 | F | 8 | 21 | 280 | 317 | 10.2 | 8.7 |
| 01-0029 | 31 | 15 | M | 6 | 44* | 387 | 330 | 7.3 | 9.0 |
| 01-0030 | 53 | unknown | F | 2 | 19 | 337 | 378 | 3.8 | 3.9 |
| 01-0031 | 63 | 38 | M | 9 | 20 | § |  |  |  |
| 01-0033 | 32 | 12 | M | 6 | 20 | 439 | 446 | 5.3 | 5.5 |
| 01-0034 | 48 | 15 | M | 6 | 36 | 513 | 456 | 2.7 | 3.1 |
| 01-0035 | 65 | 31 | M | 7 | 30 | 403 | 401 | 6.3 | 5.8 |
| 01-0036 | 25 | unknown | M | 0 | 24 | 580 | 553 | 2.1 | 2.1 |
| 01-0037 | 61 | 55 | M | 6 | 26 | 355 | 362 | 6.5 | 6.8 |
| 32-0002 | 59 | 17 | M | 5 | 23 | 643 | 462 | 3.3 | 3.5 |
| 32-0003 | 58 | 28 | M | 6 | 18 | 320 | 260 | 6.0 | 11.1 |
| 32-0004 | 51 | 8 | F | 6 | 25 | 374 | 374 | 6.4 | 5.0 |
| 32-0005 | 42 | 19 | M | 2 | 34 | 458 | 553 | 3.2 | 3.0 |
| 32-0006 | 62 | 15 | M | 6 | 35** | 495 | 246 | 7.5 | 7.3 |
| 32-0007 | 67 | 65 | F | 3 | 28 | 465 | 488 | 5.0 | 5.2 |
| 32-0008 | 57 | 25 | F | 6 | 22 | 460 | 485 | 6.3 | 6.4 |
| 32-0009 | 64 | 55 | F | 5 | 27 | 256 | 305 | 9.6 | 8.5 |
| 32-0010 | 20 | 14 | M | 3 | 20 | 460 | 481 | 2.4 | 2.6 |
| 32-0012 | 67 | 50 | F | 3 | 34 | 389 | 348 | 3.8 | 6.5 |
| 32-0013 | 42 | 31 | F | 2 | 22 | 412 | 430 | 5.6 | 6.7 |
| 32-0014 | 62 | 35 | F | 2 | 22 | 408 | 436 | 5.5 | 5.5 |
| 32-0015 | 51 | 30 | M | 6 | 20 | 274 | 195 | 10.3 | 11.9 |
| 32-0016 | 59 | 55 | M | 5 | 24 | 362 | 353 | 6.9 | 7.8 |
| 32-0017 | 42 | 6 | M | 5 | 18 | 419 | 397 | 5.7 | 5.9 |
| 32-0018 | 62 | 20 | F | 6 | 19 | 324 | 382 | 5.5 | 5.6 |
| 32-0019 | 75 | 23 | M | 7 | 24 | 352 | 252 | 8.1 | 9.7 |

Note: *FSHD2; **FSHD1+2 mosaic; §Non-ambulatory.

**Supplemental Table 2:** STIR-positive muscles by baseline STIR hyperintensity levels

|  | STIR = 1 | STIR = 2 | STIR = 3 | STIR = 4 | Total |
| --- | --- | --- | --- | --- | --- |
| Gastrocnemii | 41 | 20 | 5 | 2 | 68 |
| Vastus lateralis | 8 | 6 | 3 | 0 | 17 |
| Tibialis anterior | 5 | 7 | 5 | 0 | 17 |
| Soleus | 2 | 3 | 3 | 0 | 8 |
| Semitendinosus | 2 | 2 | 2 | 0 | 6 |
| Tibialis posterior | 0 | 1 | 2 | 0 | 3 |
| Sartorius | 1 | 1 | 0 | 0 | 2 |

**Supplemental Table 3**: LMEM results, 6MWT and Go-30-Feet regressed on visit and individual muscle fat fractions. Fat fraction coefficients correspond to IQR change increases.

| **Variable** | **6MWT** | | **Go-30-Feet** | |
| --- | --- | --- | --- | --- |
|  | **Beta [95% CI]** | **P-value** | **Beta [95% CI]** | **P-value** |
| Intercept | 496.31 [435.33, 555.93] | <.001 | 5.16 [3.64, 6.59] | <.001 |
| Follow-up vs baseline | -6.05 [-32.68, 19.81] | 0.659 | 0.2 [-0.33, 0.7] | 0.462 |
| Sartorius | 1.13 [-4.49, 6.57] | 0.718 | 0 [-0.01, 0.01] | 0.575 |
| Vastus lateralis | -0.95 [-7.08, 5.26] | 0.784 | 0 [-0.01, 0.01] | 0.541 |
| Semimembranosus | -1.83 [-4.84, 1.36] | 0.288 | 0 [0, 0] | 0.97 |
| Tibialis anterior | 2.33 [-4.47, 8.5] | 0.512 | 0 [-0.01, 0.01] | 0.643 |
| Tibialis posterior | -1.22 [-7.26, 5.07] | 0.725 | 0 [-0.01, 0] | 0.14 |
| Soleus | -4.62 [-10.86, 2.13] | 0.198 | 0 [-0.01, 0.01] | 0.91 |
| Medial gastrocnemius | -1.34 [-3.54, 0.82] | 0.278 | 0 [0, 0] | 0.309 |
| Lateral gastrocnemius | -2.92 [-8.88, 3.26] | 0.39 | 0.01 [0, 0.01] | 0.203 |
